# Circadian Timekeeping Is Disturbed in Rheumatoid Arthritis at Molecular Level

**DOI:** 10.1371/journal.pone.0054049

**Published:** 2013-01-15

**Authors:** Vesa-Petteri Kouri, Juri Olkkonen, Emilia Kaivosoja, Mari Ainola, Juuso Juhila, Iiris Hovatta, Yrjö T. Konttinen, Jami Mandelin

**Affiliations:** 1 Department of Medicine, Institute of Clinical Medicine, University of Helsinki, Helsinki, Finland; 2 Department of Anatomy, Institute of Biomedicine, University of Helsinki, Helsinki, Finland; 3 Department of Medicine/invärtes medicin, Helsinki University Central Hospital, Helsinki, Finland; 4 Research Program of Molecular Neurology and Department of Medical Genetics, Haartman Institute, University of Helsinki, Helsinki, Finland; 5 Department of Mental Health and Substance Abuse Services, National Institute for Health and Welfare, Helsinki, Finland; 6 ORTON Orthopedic Hospital of the ORTON Foundation, Helsinki, Finland; 7 COXA Hospital for Joint Replacement, Tampere, Finland; University Hospital Jena, Germany

## Abstract

**Introduction:**

Patients with rheumatoid arthritis (RA) have disturbances in the hypothalamic-pituitary-adrenal (HPA) axis. These are reflected in altered circadian rhythm of circulating serum cortisol, melatonin and IL-6 levels and in chronic fatigue. We hypothesized that the molecular machinery responsible for the circadian timekeeping is perturbed in RA. The aim of this study was to investigate the expression of circadian clock in RA.

**Methods:**

Gene expression of thirteen clock genes was analyzed in the synovial membrane of RA and control osteoarthritis (OA) patients. BMAL1 protein was detected using immunohistochemistry. Cell autonomous clock oscillation was started in RA and OA synovial fibroblasts using serum shock. The effect of pro-inflammatory stimulus on clock gene expression in synovial fibroblasts was studied using IL-6 and TNF-α.

**Results:**

Gene expression analysis disclosed disconcerted circadian timekeeping and immunohistochemistry revealed strong cytoplasmic localization of BMAL1 in RA patients. Perturbed circadian timekeeping is at least in part inflammation independent and cell autonomous, because RA synovial fibroblasts display altered circadian expression of several clock components, and perturbed circadian production of IL-6 and IL-1β after clock resetting. However, inflammatory stimulus disturbs the rhythm in cultured fibroblasts. Throughout the experiments *ARNTL2* and *NPAS2* appeared to be the most affected clock genes in human immune-inflammatory conditions.

**Conclusion:**

We conclude that the molecular machinery controlling the circadian rhythm is disturbed in RA patients.

## Introduction

Endogenous circadian rhythm is maintained by the central circadian timekeeper, the suprachiasmatic nuclei of the hypothalamus. The clock machinery is composed of heterodimeric transcription factors and transcription regulatory proteins. The expression of these factors oscillates rhythmically over 24-hour cycles [1]–[3]. The rhythm of the central master clock is transferred to cells in the peripheral organs through hormonal and neuronal connections [2], [4]. The de-synchronization of endogenous and geophysical time leads to fatigue as in jet lag. Altered circadian rhythm can cause e.g., cancer and obesity [5], [6].

The molecular core of the circadian machinery is composed of transcription factors Aryl hydrocarbon Receptor Nuclear Translocator-Like (*ARNTL/BMAL1*) and Circadian Locomotor Output Cycles Kaput (*CLOCK*), which heterodimerize and activate transcription from E-box elements located in the promoters of clock controlled genes. Their paralogs *ARNTL2* (*BMAL2*) and Neuronal PAS domain-containing protein 2 (*NPAS2*), respectively, may functionally replace them. These transcription factors drive the expression of clock output genes, for example albumin D-box binding protein (*DBP*) but also their own repressors cryptochrome (*CRY1*, *CRY2*) and period (*PER1*, *PER2* and *PER3)* proteins. CRY and PER proteins form heterodimers and translocate to nucleus, in which they inhibit the function of DNA bound BMAL1:CLOCK heterodimer. Thus, CRY and PER proteins inhibit their own expression but also the expression of other clock controlled genes composing a negative regulatory limb of the circadian clock. RAR-related Orphan Receptor proteins RORs and REV-ERBs effect on the positive limb of circadian machinery. RORs bind into the promoter region and drive the expression of *BMAL1*. By competing of this binding site REV-ERBs inhibit ROR activity. This network of feedback loops affecting on the positive and negative sites around the core drives the usually extremely accurate self-sustained rhythmic expression of the circadian clock genes. Basic helix–loop–helix (bHLH) transcription factors *BHLHE40* (*DEC1*) and *BHLHE41* (*DEC2*) directly inhibit BMAL1:CLOCK by competing in binding of E-box elements in DNA. Thus, they form still another layer of the control mechanisms. This complex system of transcriptional regulation forms the molecular clockwork of the circadian timekeeper and creates the rhythmic expression of its output genes [1], [2], [4].

Rheumatoid arthritis (RA; MIM 180300) is a chronic inflammatory joint disease, a progressive destructive and debilitating arthritis caused by an invasive infiltrate composed of inflammatory cells and synoviocytes. Patients suffering from RA as well as other autoimmune diseases display blunted hypothalamic-pituitary-adrenal (HPA) axis reactions to inflammation [7], altered rhythm of circulating cortisol, melatonin and IL-6 levels [8], [9]. Remarkably, up to 80% of patients experience chronic fatigue [10], [11].

The function of T- and B-cells is perturbed in RA. This is seen e.g., in autoantibody production and in the fact that therapeutic targeting of either T- or B-cells is an effective treatment for RA. Interestingly, the same molecules that function in the intact clock controlling circadian rhythm e.g., rhythmic cortisol production [12] also control the development and function of immune cells. For example, *Dec1* deficient animals develop systemic autoimmunity [13], *Dec2* affects the development of Th2 cells [14], *Rorc* the development of Th17 cells [15] and *Rora* is a negative regulator of NF-κB signaling pathway [16].

It also appears that there are bidirectional links between the circadian time keeping and TNF-α production. For example, *Cry1/Cry2* deficiency in mice affects TNF-α production and exacerbates arthritis [17]. Conversely, TNF-α can alter the expression of clock genes and inflammation modifies the endogenous rhythm [18], [19].

Because RA patients suffer from chronic fatigue, have immune cell dysfunction, and pathogenic production of TNF-α, all of which are affected by the molecular clock, we hypothesized that the circadian timekeeping is perturbed in RA. Thus, we analyzed the function of the circadian clock in RA patients and compared that to the function of clock in osteoarthritis (OA) patients, who have cartilage deterioration without autoimmunity and chronic fatigue. By doing so, we revealed clearly abnormal molecular clockwork in the synovial membrane tissue of the patients as well as in the cells derived from the synovial membrane.

## Methods

### Subjects

The research plan was approved by the ethical committee of the Helsinki University Central Hospital. Guidelines of the Declaration of Helsinki were followed. RA patients fulfilled the 1987 revised criteria of the American College of Rheumatology. Tissue samples (n = 10 for both RA and OA patients) were taken at 10 a.m. ±2 h during synovectomy or operation for total joint replacement. Samples were divided so that one half was formalin fixed and embedded in paraffin and the other half was immediately snap-frozen in isopentane precooled by dry ice and stored at −70°C.

### Immunohistochemistry

Serial 3 µm thick paraffin tissue sections from the formalin fixed synovial membrane samples were stained using Bond MaX fully automated IHC and ISH Staining System (Leica Microsystems) using programs and Bond reagents for Dewax solution for deparaffinization, Epitope Retrieval solution 1 for antigen retrieval and Bond Polymer Refine kit for antigen detection. Briefly, slides were deparaffinised followed by one wash in absolute ethanol. Antigens were retrieved following endogenous peroxidase quenching. Sections were incubated in 1 µg/ml rabbit anti-MOP3 (anti-Bmal1; AB4140, Chemicon). Rabbit IgG at the same concentration was used for negative staining control. After secondary antibody containing the HRP polymer, color was developed using H_2_O_2_ and DAB. Between each step slides were washed at least three times. Finally, slides were dehydrated, cleared and coverslips were mounted using Mountex (Histolab).

### RNA Isolation, cDNA Synthesis and Quantitative Real-time PCR

RNA was isolated using RNeasy Mini Kit according to the manufacturer’s instructions. RNA concentrations were measured using NanoDrop ND-1000 instrument (Thermo Fisher Scientific). The cDNA synthesis was performed using 500 ng of total RNA and iScript™cDNA Synthesis Kit (Bio-Rad) in a 20 µl reaction volume. Quantitative real-time PCR was performed from 1∶5 diluted cDNA in iQ™ SYBR® Green Supermix (Bio-Rad) using gene specific primers (Table 1) in 20 µl reaction volume. The PCR was performed in iQ5 real-time PCR detection system (Bio-Rad).

**Table 1:** Primers.

Gene	GeneBank Accession	5′ Primer	3′ Primer	Length
BMAL1	NM_001178	CTGGAGAAGGTGGCCCAAAGAG	CCACTGGAAGGAATGTCTGGAGTC	250
ARNTL2	NM_020183	GCTAGAGGCTACCAGGCAAAACC	GGTCCACTGGATGTCACTGAAGTC	193
CLOCK	NM_004898	TTCTGCCTCTTCTCGGAGTTCAAG	CCTGGGTGGAGTGCTCGTATC	103
NPAS2	NM_002518	CTTCCCTGCCTCCCAACCATC	GGTCCCTGGCTGTTGTGAGTAG	151
BHLHE40	NM_003670	TCAGCAGCAGCAGAAAATCATTGC	GTGGGTGACAAGCTGCGAAGAC	187
BHLHE41	NM_030762	TGCTTTACAGAATGGGGAGCGATC	CCCTGGGTGTCCAGCTCTCAAAC	134
PER1	NM_002616	CTCCAATCAGGACGCACTTTC	GCTGCCAAAGTATTTGCTTGTG	211
PER2	NM_022817	TGTAGGGGCGGACTGCAAAC	TGCTGGTATGACTTGTGTCACTAC	251
PER3	NM_016831	TGAAGAATCCATCCCATCCTACTG	TATACTGCTGTCGCTGCTTCC	218
CRY1	NM_004075	TCTGGCATCAGTACCTTCTAATCC	CTGTGTGTCCTCTTCCTGACTAG	226
CRY2	NM_021117	GGTGAAGAACTCAGCAAACGG	ACACACATGCTCGCTCTATCTC	189
RORA	NM_134262.2	CCAGCCCCGACGTCTTCAAAT	GCCATGAGCGATCTGCTGACA	150
RORB	NM_006914.3	ACCGTTGCCAACACTGCCGA	GCTGGTGCTTCTGCACCTCA	126
RORC	NM_001001523.1	GGGCTGCAGCGAGCTCATCA	TCTCTTGGAGCCCTGGCCGA	134
NR1D1	NM_021724	CTTGGCTGCCCAGCGTCATAAC	CCAGATCTCCTGCACCGTTCG	274
DBP	NM_001352	CTTAAGCCCCAGCCAATCATGAAG	CCGCCCGCACCGATATCTG	160
IL-1β	NM_000576	TGGCAATGAGGATGACTTGT	GGAAAGAAGGTGCTCAGGTC	237
IL-6	NM_000600.3	AGGAGACTTGCCTGGTGAAA	GAGGTGCCCATGCTACATTT	329
β-act	NM_001101.3	TCACCCACACTGTGCCCATCTACGA	CAGCGGAACCGCTCATTGCCAATGG	295
PBGD	NM_000190.3	ACATGCCCTGGAGAAGAATG	AGATGCGGGAACTTTCTCTG	237
RPLP0	NM_001002	GGCGACCTGGAAGTCCAACT	CCATCAGCACCACAGCCTTC	149

### Cell Culture

Synovial fibroblasts from RA (n = 3) and OA (n = 3) patients were established using explant culture method. Tissue samples were cut into pieces with a sterile scalpel in a laminar flow hood. The explants were left overnight in RPMI-1640 medium (Lonza) containing 10% fetal bovine serum (Lonza) with 1000 U/ml penicillin and 0.1 mg/ml streptomycin (10×) solution. The next day, the media were changed to basal RPMI with 10% FBS media and 100 U penicillin and 0.1 mg streptomycin (1× solution). The medium was changed twice a week for 3 weeks. After roughly 80% monolayer confluence was reached, the explants were removed, and the cells were subcultured 1∶3 until confluent. Passages 2–4 were used for subsequent experiments.

### Clock Resetting with Serum Shock

The clock resetting in RA and OA fibroblasts was performed as reported earlier [20]. Briefly, cells were cultured in RPMI including supplements until ready for the serum shock. For the serum shock 150 000 cells were plated on 6-well plates. The cells were grown to confluence. At TIME = -2, the medium was replaced to RPMI containing penicillin/streptomycin and 50% horse serum (GIBCO). Samples to determine baseline were also collected at this point. After 2 hours, the medium was replaced with serum-free RPMI containing the antibiotics (TIME = 0). Exactly at the indicated times, the wells were washed with PBS and lyzed with 350 µl RLT lysis buffer (Qiagen). The lysates were stored at −70°C until RNA isolation was performed.

### TNF-α and IL-6 Stimulation

OA fibroblasts were cultured in RPMI including supplements until ready for the stimulations. For the stimulations 80 000 cells were plated on 12-well plates. The cells were grown to confluence. Before stimulations the cells were synchronized with 18 hours serum starvation using RPMI containing penicillin/streptomycin and 1% fetal bovine serum. At TIME = 0, the media were replaced with RPMI media containing penicillin/streptomycin, 1% fetal bovine serum and TNF-α (10 ng/ml; R&D Systems), IL-6 (10 ng/ml; R&D Systems) or no cytokines (negative control). At the indicated times, the wells were washed with PBS and lyzed with 350 µl RLT lysis buffer (Qiagen). The lysates were stored at −70°C until RNA isolation was performed.

### Statistical Analysis

The data from tissues were analyzed using Student’s t-test and Pearson correlation test with SPSS 15.0 for Windows. The data from clock reset experiment were fitted to a sine wave equation, y(x) = BaseLine+Amplitude sin (frequency x+PhaseShift) in Matlab (The MathWorks Inc., Natick, MA, USA). The frequency was fixed to 24 hours. From IL-1β, IL-6 and Per1, the first time point (TIME = 0) was excluded from the fit, to avoid errors generated by the initial high expression peak caused by the stimulus. A Student’s t-test was applied to determine, whether or not the phase, baseline (general expression level) or amplitude (peak expression) was shifted between RA and OA samples.

## Results

### Clock Gene Expression is Disturbed in RA Synovium

**Figure 1:**
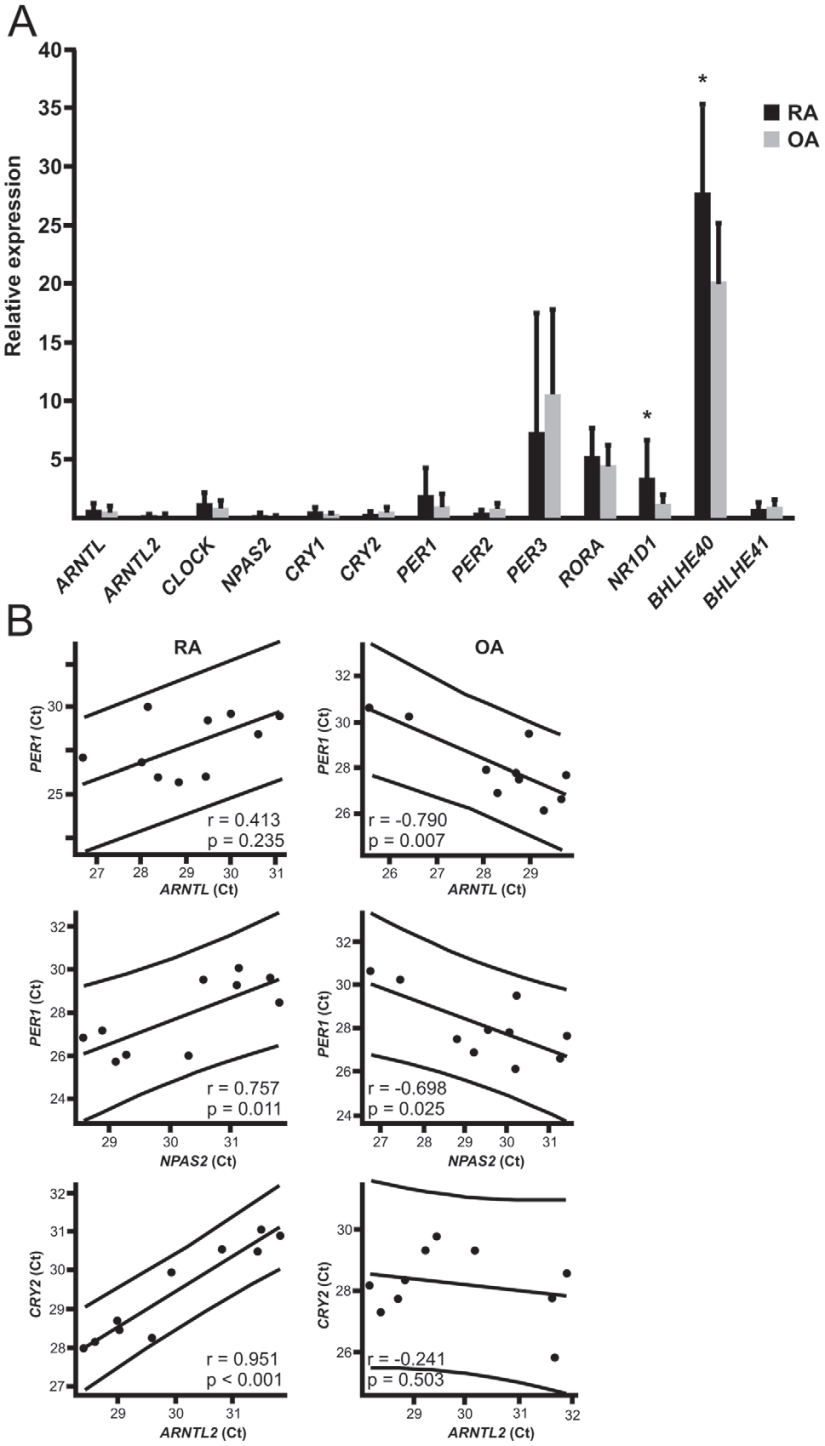
The expression of circadian genes in RA and OA patients. A. Synovial membrane samples were collected from RA and OA patients (n = 10 for each) and analyzed for circadian gene expression. The relative expression of each gene is expressed as mean ± SD. B. The expression of *BMAL1* and *PER1* should be in anti-phase. Pearson correlation and the linear fit for the expression of *BMAL1* and *PER1* in the synovial membrane of RA patients reveals that high expression of *BMAL1* correlates with high *PER1* expression and *vice versa*, low *BMAL1* expression correlates with low *PER1* expression. In contrast to synovium of RA patients, the high expression of *BMAL1* was associated with low *PER1* expression and conversely, low *BMAL1* expression was coupled with high *PER1* expression in the synovial membrane of OA patients. Similar findings were observed with *NPAS2* and *PER1* as well as with *ARNTL2* and *CRY2*. Each dot indicates the Ct value of *BMAL1*, *NPAS2* or *ARNTL2* in the x-axis and *PER1* or *CRY2* in the y-axis in the synovial membrane sample of an individual patient. *p<0.05.

Driven by our hypothesis that the circadian timekeeping is perturbed in RA, we analyzed expression levels of clock genes in the synovial membrane of RA patients in comparison to OA patients. The expression levels of *BHLHE40* and *NR1D1* (*REV-ERBα*) were significantly increased in RA synovium compared to OA synovium (Figure 1a).

To overcome the variation of sampling time between individuals we reasoned that the best indication of the subjective (endogenous) time would be the correlation of the expression of *BMAL1* and *PER1*, which are normally in antiphase against each other. Due to the negative feedback loop, the peak expression of *BMAL1* and *PER1* should definitely not occur at the same time. Indeed, this was true in the control synovial membranes obtained from OA patients: high *BMAL1* expression coexisted with low *PER1* expression and *vice versa* (Figure 1). RA patients, in contrast, did not have this kind of anti-phase *BMAL1* and *PER1* expression (Figure 1). Similarly, high *ARNTL2* and *NPAS2* expression occurred simultaneously with the inhibitory clock components only in RA. Most striking was the significant positive correlation of *NPAS2* and *PER1* expression in RA (Figure 1), while it as expected was significantly negative in OA (Figure 1). Further, *ARNTL2* expression correlated positively with the expression of *CRY1* (.715; p<0.05), *CRY2* (.951; p<0.001), *PER2* (.862; p<0.01), *BHLHE40* (.936; p<0.001) and *BHLHE41* (.785; p<0.01) only in RA.

### Ectopic Sub-cellular BMAL1 Protein Localization in RA Synovium

**Figure 2:**
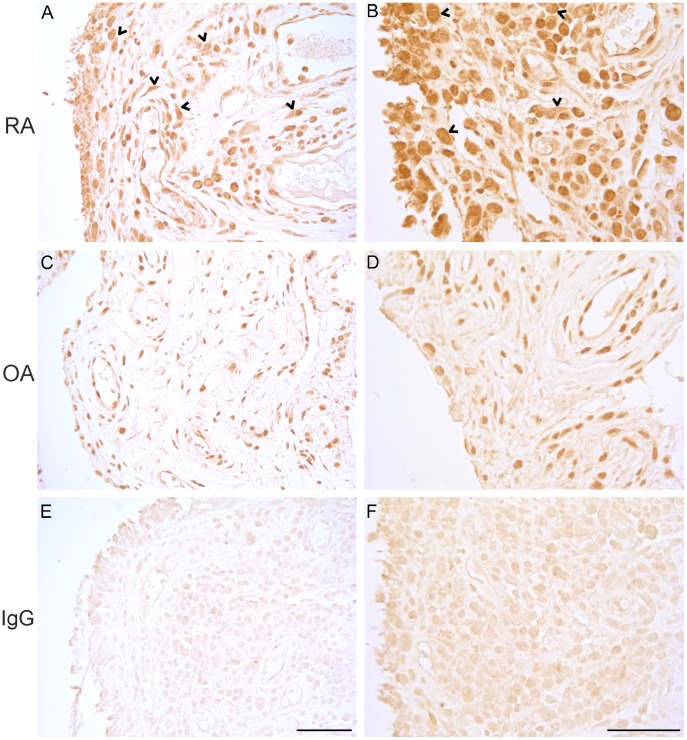
Localization of BMAL1 protein in RA and OA synovium. Tissue sections were incubated in rabbit anti-BMAL1 (anti-MOP3). In RA (A and B), relatively strong immunoreactivity was detected in the nuclei (arrows) of the immune cells and fibroblasts, but even stronger immunoreactivity was observed in the cytoplasm of the cells (arrowheads). In contrast to RA, BMAL1 protein was localized only in the nuclei (arrows) of OA synovium (C and D). Irrelevant rabbit IgG was used to confirm the specificity of the BMAL1 antibody (E and F). Scale bar 100 µm.

After revealing that the expression of clock genes is disturbed in the RA patients, we sought for confirmation to this observation on the protein level. We used immunohistochemistry to localize the limiting core clock component BMAL1 in the synovial tissues of RA and OA patients. The intense cytoplasmic BMAL1 protein staining in RA tissues draws attention to another major aberration. Not only are the phases of *ARNTL*s and *NPAS2* relative to *CRY*s and *PER*s disturbed in RA, but this is also accompanied by ectopic localization of a core clock component (Figure 2).

### Cell Autonomous Defect of Clock Function in RA

**Figure 3:**
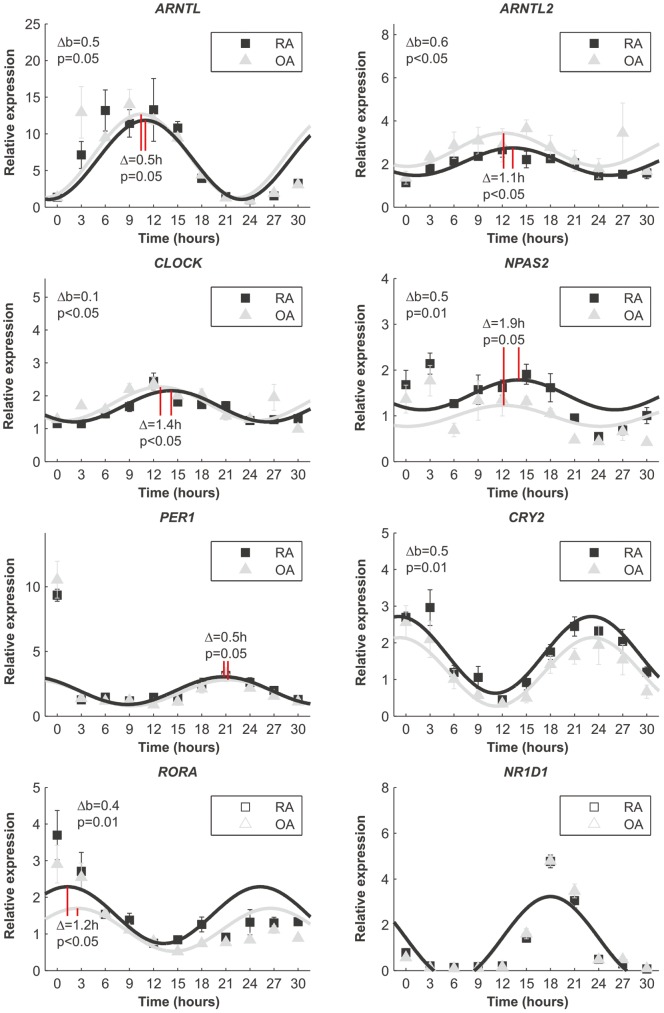
Relative clock gene expression over time after serum shocked primary fibroblasts. Fitted sin graphs of the expression and mean ± SD of the mean from three individual patients per group are shown. The time of serum removal is 0 in the graphs and the relative expression is normalized to the expression before serum (at time = −2). Δb indicates the general difference in the expression i.e., the baseline of the expression. Phase differences are marked as red solid lines and the difference is indicated in hours.

To rule out the possibility that inflammation, medication or some endocrine factors in the patients is the reason for the perturbed expression of circadian genes in RA synovium, we induced clock resetting with serum [20] in synovial fibroblasts isolated from RA and OA tissue samples. Immediately after resetting the expression pattern of *BMAL1* was delayed 0.5 hours (p = 0.05) in RA fibroblasts compared to OA fibroblasts, i.e. the expression of *BMAL1* in RA cells peaked 30 minutes later that OA cells. In addition to this, the expression level of *BMAL1* in RA cells never reached the level of OA (p = 0.05). The expression peak of *ARNTL2* was delayed 1.1 hours (p<0.05) and the level was throughout the experiment significantly lower (p<0.05) in RA cells compared to OA cells. Similarly, *CLOCK* expression was delayed 1.4 hours (p<0.05) in RA fibroblasts and the relative expression was constantly lower (p<0.05) in RA cells and *NPAS2* expression was delayed 1.9 hours (p = 0.05) but was constantly higher in RA fibroblast (p = 0.01) than in OA fibroblasts. Based on the delayed expression of the core clock, one would expect that the expression of clock driven circadian genes would lag in RA cells compared to OA cells. In contrast, the expression of *PER1* increased 0.5 hours earlier (p = 0.05), and the expression of *CRY2* was constantly higher in RA than OA cells after serum shock. Also *CRY1* expression increased earlier in RA cells than in OA cells, indicating that expression of the negative feedback loop is activated faster in RA than in OA. On the positive limb of the circadian machinery, *RORA* expression was 1.2 hours (p<0.05) ahead and constantly higher (p = 0.01) in RA cells than in OA cells, and *NR1D1* expression was exactly the same in both cell types (Figure 3). *RORB* and *RORC* were not expressed.

### Altered Circadian IL-1β and IL-6 Expression in RA Fibroblasts

**Figure 4:**
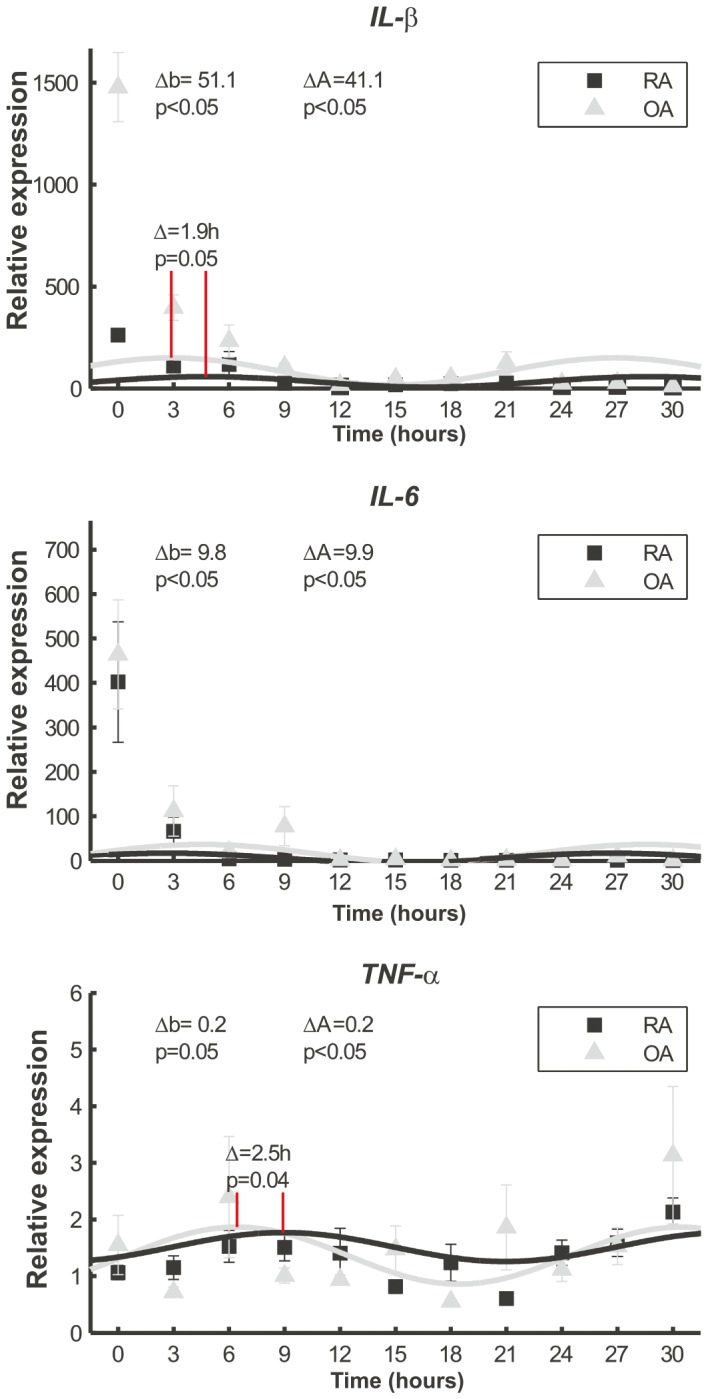
The oscillation of *IL-1β*, *IL-6* and *TNF-α* in RA and OA cells after serum shock. Relative gene expression over time is displayed. Fibroblasts derived from RA synovium display weaker rhythmic expression of *IL-1β*, *IL-6* and *TNF-α* after clock resetting than cells from OA synovium. Fitted sin graphs of the expression and mean ± SD of the mean from three individual patients per group is shown. The time of serum removal is 0 in the graphs and the relative expression is normalized to the expression before serum (at time = −2). Δb indicates the general difference in the expression i.e., the baseline of the expression. ΔA indicates the differences in the peak amplitude. Phase differences are marked as red solid lines and the difference is indicated in hours.

RA patients display altered circadian hormonal and cytokine profiles. For example, the circulating concentration of IL-6 in RA patients differs from that of healthy individuals [8]. We tested the possibility that the perturbed clock function is also reflected in disturbed *IL-6* expression after clock resetting. Fibroblasts derived from RA displayed significantly weaker rhythmic expression of *IL-6* after clock resetting than cells from OA synovium (Figure 4). Both the general expression as well as the peak expression was higher in OA cells (p<0.05 for both). Because of this observation, we analyzed if this is the case also for *IL-1β* or *TNF-α* expression, which are also rhythmically produced pro-inflammatory cytokines [21], [22]. Similar to *IL-6* expression, RA fibroblasts displayed significantly weaker rhythmic expression of *IL-1β* than OA fibroblasts (p<0.05) Also *TNF-α* expression was rhythmic over time. In contrast to the expression of *IL-1β* and *IL-6*, the general expression level of *TNF-α* was higher (p = 0.05) in RA cells than in OA cells. Like in the expression of *IL-1β*, both the phase (p<0.05) and the amplitude (p<0.05) in *TNF-α* expression were different between the cells derived from RA and OA synovium (Figure 4) confirming the near absent and misaligned self-sustained rhythmic expression of pro-inflammatory cytokines in RA cells.

### Inflammation Changes the Expression of Clock Genes In Vitro

**Figure 5:**
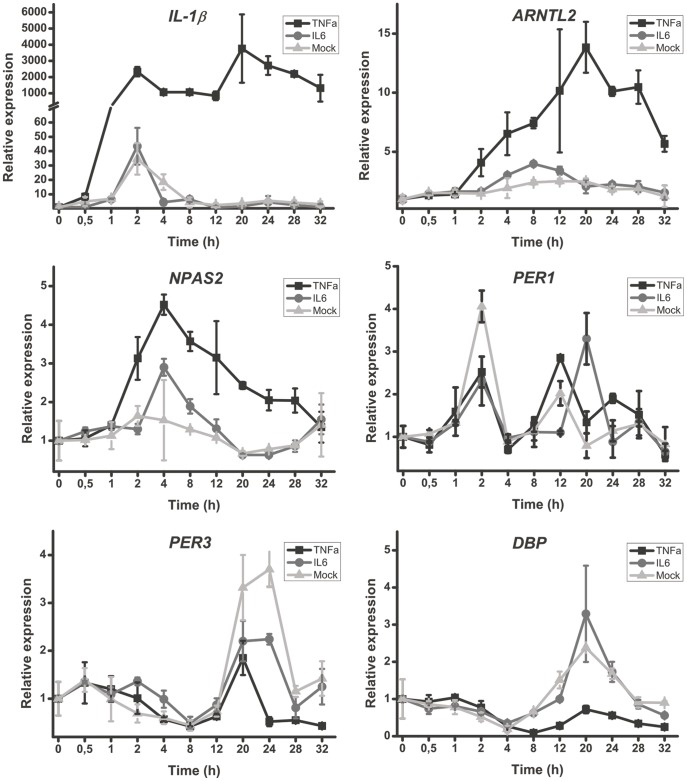
TNF-α increased *ARNTL2* and *NPAS2*, and suppresses *PER3* and *DBP* expression but IL-6 did not. Relative gene expression over time is displayed. Representative data from one experiment is shown. The experiment was repeated five times using fibroblasts from different OA patients. The time of TNF-α or IL-6 addition is 0 in the graphs.

Due to the bidirectionally maintained control of clock and inflammation, we investigated the effect TNF-α on human fibroblasts. A strong response in the expression of *IL-1β* (Figure 5) was evident confirming the effect of TNF-α on the cells. The most significantly affected clock genes after stimulation with TNF-α were *ARNTL2* and *NPAS2*. Unlike earlier observations in mouse cells [18], TNF-α did not affect the expression of *PER1*. However, the inhibitory (anti-phase) effect of TNF-α on the expression of clock controlled genes *DBP* and *PER3* was clear (Figure 5). IL-6 did not markedly alter the expression of clock genes.

## Discussion

Abnormal fatigue, morning stiffness and altered circadian hormone and cytokine production suggest that the internal neuroimmunoendocrine milieu and the activity of daily living may be desynchronized in RA patients [7], [8], [10] i.e., the body is adjusted for activity (subjective day) during sleep (geophysical night), but meets the challenges of the day when geared for sleep and rest. Central molecular clock in the hypothalamus normally synchronizes body functions and entrains the circadian clocks in all cells of the peripheral tissues [2], [4]. According to our findings the clock is dysfunctional in the joints of RA patients. Our results suggest that inflammation may initially disturb circadian timekeeping and that a cell autonomous defect prevents accurate clock function in the synovial fibroblasts of RA patients.

The variation in the expression of circadian genes at a defined and very narrow time point used for sample collection was evident between different individuals. Due to the characteristic oscillation of the expression of clock genes [1]–[3] leading to individual sleeping habits of each subject this was expected. To overcome the problem, we utilized the characteristic property of the clock and analyzed the endogenous (subjective) time using the expected anti-phase expression of inhibitory circadian genes in RA synovium and in OA synovium, i.e. checked the circadian time. This anti-phase expression of inhibitory circadian components, which forms the very basis for the circadian timekeeping, was totally lost in RA synovium i.e., the endogenous circadian time is clearly perturbed in the synovial membrane tissue of RA patients.

The perturbed expression of the machinery is also reflected in the amount and sub-cellular localization of BMAL1 protein. Apparently, there is much higher levels of BMAL1 protein in RA than OA while at RNA levels the difference is not significant. Of the core clock genes the expression of *BMAL1* varies the most during the day. Due to this oscillation of the expression, however, we may have not detected the highest differences in the gene expression. The most dramatic differences in the gene expression may well be seen at different time points whereas the amount of protein in the tissue may reflect the differences in the expression or different kinetics in the posttranslational mechanisms that regulate the degradation of the core clock proteins [23], [24]. This may well explain why the gene expression and protein amounts do not correlate. However, the result reinforces the finding of a disturbed clock in RA tissue.

RA patients suffer from chronic inflammation and altered glucocorticoid signaling, both of which affect the circadian timekeeping effectively [18], [25]. Thus, these factors or medication might explain altered clock gene expression in the synovium of RA patients. This possibility was excluded using cultured synovial fibroblast derived from RA and OA patients. Clock gene oscillation in fibroblasts can be started with serum, which activates several intracellular pathways. Due to the intrinsic character of the clock the cells maintain the rhythm several days without any external time cues and further activation of the signaling pathways [20]. This phenomenon thus reflects the normal function of the endogenous timekeeping molecular machinery. Using this system we observed near two-hour phase differences between RA and OA in the circadian gene expression already short after clock resetting. This is remarkable, because normally the self-sustained rhythm varies only 0.32 hours per day [26] and patients from whom these fibroblasts were isolated were randomly selected i.e., they were not selected by chronotype or any other factor that may impact the results. Thus, our results most likely reflect true differences between RA and OA patients. Importantly, the expression of *PER1*, for example, increased equally in RA and OA cells immediately after clock resetting, but the self-sustained rhythm was divergent. This excludes the effect of the initial serum shock, inflammation or other endocrine factors as the sole reasons for the disturbed clock gene expression in RA synovium and underlines a primary, cell autonomous defect in the control of the biorhythm in RA, i.e. a defect in the circadian clockwork itself.

Because RORs drive the expression of *BMAL1* [27], [28], lag of *BMAL1* expression suggests delayed or diminished expression of *ROR* genes or a low ratio between the expression of *ROR* genes and their competitor *REV-ERBα* in RA cells. However, early and increased *RORA* expression in RA cells and equal *REV-ERBα* expression in RA and OA cells exclude this possibility. The expression of *ARNTL2* and *NPAS2* were also different in RA and OA. This is particularly important based on the fact that *ARNTL2* and *NPAS2,* not *PER1,* react to inflammation in human fibroblasts and some mouse tissues (data not shown).

RA patients have HPA-axis dysfunction in response to inflammation [7], [8]. Due to the bidirectional links between inflammation and clock, we wanted to investigate the effect of inflammation on clock gene expression. IL-6 did not affect significantly the expression of clock genes. Thus, the purpose of robust oscillation of IL-6 levels in the blood most likely is not to synchronize peripheral clocks. However, TNF-α affected clock gene expression in the cultured synovial fibroblasts, indicating that inflammation can disturb normal circadian rhythm rapidly in the peripheral tissues.

Accurate circadian clock allows orchestrated proactive rather than reactive bodily functions. The data presented here clearly show that RA patients have a deficiency in the function of the clock. It must be emphasized that it is not the synovium or synovial fibroblasts that may cause these disturbances. Rather this cell autonomous phenomenon reflects the behavior of all molecular clocks in the body including the central clock, because circadian clock has virtually the same molecular makeup in the central timekeeper and different peripheral cells [29]. Based on this, our *in vitro* results suggest that RA patients, despite obtaining resetting stimuli every day, tend to lose the rhythm relatively fast due to a cell autonomous defect in their circadian clock. This may explain why the patients experience the fatigue as if they would be in a constant jet lag. The clock not only controls the circadian rhythm but also affects the development and function of immune cells. Thus, the immunological, neuronal and endocrine problems observed in the RA patients may be caused by cell autonomous dysfunction of the clock. This is further reinforced by the fact that novel biotherapies (anti-TNF, anti-B cell, anti T-cell, or anti-IL-6) have only limited impact on chronic fatigue in RA [30] at least partly excluding the possibility that inflammatory factors directly cause this severe problem in RA. Notably, *ARNTL2* and *NPAS2* are the clock genes that throughout different experiments were the most disturbed.

### Conclusion

Herein, we show that RA patients display cell autonomous disconcerted circadian timekeeping. Throughout the analysis *ARNTL2* and *NPAS2* were the genes revealed to be most associated with human inflammatory conditions. Strikingly, *PER1* did not react to inflammatory stimulation in human fibroblasts. It appears that RA patients may experience the fatigue as if they would be in constant jet lag. Thus, chronotherapy that takes this altered rhythm into account and/or tries to correct it could be beneficial for the patients.
